# Effects of Si/C Ratio on the Phase Composition of Si-C-N Powders Synthesized by Carbonitriding

**DOI:** 10.3390/ma13020346

**Published:** 2020-01-12

**Authors:** Cong Zhang, Ling Qu, Wenjie Yuan

**Affiliations:** 1 The State Key Laboratory of Refractories and Metallurgy, Wuhan University of Science and Technology, Wuhan 430081, China; jidechizaofan1539@163.com (C.Z.); quling_cyxs@163.com (L.Q.); 2 National-Provincial Joint Engineering Research Center of High Temperature Materials and Lining Technology, Wuhan University of Science and Technology, Wuhan 430081, China

**Keywords:** Si-C-N powders, phase composition, carbonitriding

## Abstract

Si-C-N based materials possess interesting properties such as high hardness and oxidation resistance. The compacts of silicon and cornstarch with different Si/C ratios were subjected to carbonitriding at 1350–1550 °C. Reaction products were characterized by X-ray powder diffractometer (XRD), X-ray photoelectron spectroscopy (XPS), scanning electron microscopy (SEM) and transmission electron microscope (TEM). The effects of Si/C ratio on the phase composition of Si-C-N powders were investigated. The results revealed that the Si/C ratio played a crucial role on the formation of crystalline silicon carbonitride (SiCN) and the phase composition of Si-C-N powders. It was demonstrated that liquid silicon is an important medium and reaction site for the introduction of nitrogen, so the Si content in reactants has affected the N content in the product. On the other hand, carbon participates in the carbonization of Si_3_N_4_ and the formation of SiC. The contents of C-N bond and SiCN in the products are carbon content-dependent. Combining the above two aspects, the maximum yield of SiCN can be achieved with the Si/C ratio of 1:1 to 1:1.5.

## 1. Introduction

Compared with the properties of SiC and Si_3_N_4_, Si-C-N materials exhibit more promising features including high strength, high hardness and good oxidation resistance [1,2,3]. In addition, Si-C-N materials are of great technological importance due to their novel optical [4], photoelectric [5], electrical [6,7] and electromagnetic properties [8,9,10]. The synthesis of covalent materials such as non-oxides is difficult because of low diffusion coefficients. Conventionally, ternary SiCN materials are prepared by the pyrolysis of preceramic polymers [11], the magnetron sputtering [12] and the vapor deposition [13]. In the polymer-derived ceramic (PDC) route, amorphous Si-C-N compounds were made of the polymer precursors by the thermally induced ceramisation [14]. Other elements such as Al [15,16,17], Ti [18,19] and B [20,21] can be introduced through a precursor for synthesis [22,23,24,25], which can alter properties of SiCN based materials [26]. For SiCN materials prepared by Microwave CVD, three compounds including SiH_4_, CH_4_ and NH_3_ were used [27], and NH_3_ could be replaced by N_2_ [28]. Amorphous SiCN films can be prepared on Si substrates by nitrogen ion-assisted pulsed-laser ablation of a SiC target [29]. The fabrication of SiCN composite ceramics by chemical vapor infiltration (CVI) was exceedingly time consuming [30], but the dense bulk could be obtained by CVI [31].

According to first principle calculations, Si-C-N compounds can be divided into silicon rich forms of Si_2_CN_4_ and carbon rich forms of SiC_2_N_4_ [32,33]. Due to the change of chemical bonds, the hardness and other properties of SiCN depend on element contents [34,35]. It was found that the bulk carbon-rich SiCN ceramics possessed an improved thermal stability against crystallization compared to their powder analogues [36]. The chemical composition of SiCN films was controlled by changing the flow rate of N_2_ in CVD/PVD processes [37,38]. The increase of the N_2_/Ar flow ratio favored to the nitrogen incorporation into the films and the formation of Si-N bond, and restrained the formation of C-C, C-Si and N-C bonds [39]. The deposition product tended to be graphite when the N/Si ratio was low, and inclined to be amorphous SiCN and Si_3_N_4_ when the N/Si ratio was high [8]. The crystallization temperature of amorphous SiCN derived from the organometallic precursor increased consistently with the C/Si atomic ratio [40].

In previous work, a facile route to synthesize crystalline SiCN by using silicon and cornstarch powders as the starting materials was presented [41]. Crystalline SiCN formed at different temperatures was characterized by different methods. It was demonstrated that the oxidation of as-received SiCN powders was better than that of SiC and Si_3_N_4_. Amorphous PDC-SiCN ceramics crystallized and decomposed at high temperatures [42], however, SiCN synthesized by carbonitriding was expected to have good thermal stability. But effects of raw material’s ratio on the carbonitriding reaction were not clear yet. Low-cost large-scale preparation of mono-phase SiCN was not achieved. In this work, Si-C-N powders were synthesized by carbonitriding of silicon/cornstarch compacts with various ratios. Effects of Si/C ratio on the phase composition of Si-C-N powders synthesized by carbonitriding were investigated in order to obtain the mono-phase SiCN. The changes of phase composition, chemical bonding and morphology for the as-received products were studied.

## 2. Materials and Methods 

The silicon powders with the particle size less than 74 µm and the purity of 99.99% and the cornstarch as a source of carbon were mixed proportionally (as listed in Table 1). The mass percentage of residual carbon in cornstarch after pyrolysis at high temperatures was estimated as 20% [43]. Then the powder mixture was uniaxially pressed at 250 MPa. The compacts with a diameter of 20 mm were obtained. The nitridation process was carried out in nitrogen (99.999% purity) as follows: (1) heat up to 1350 °C at a rate of 3 °C/min followed by soaking for 1 h; (2) sequentially heat up to 1450 and 1550 °C at a rate of 5 °C/min and soaked for 3 h. The products were milled into fine powders for the characterization.

The crystalline phases in the as-received products were identified by X-ray powder diffractometer (XRD, X′pert Pro MPD, Philips, Almelo, The Netherlands) with Cu Kα radiation (λ = 0.15406 nm) operated at 40 kV and 40 mA. The chemical composition and bonding states of samples were determined by X-ray photoelectron spectroscopy (XPS, PHI Quantera II, ULVCA-PHI, Kanagawa, Japan) with the energy step size of 0.1 eV and spot size of 200 μm. Al Kα radiation of 1486.6 eV was used as the X-ray source. Scanning electron microscopy (SEM, VEGA II, TESCAN, Brno, Czech Republic) with energy dispersive X-ray spectroscopy (EDS, INCA X-act, Oxford Instruments, Oxford, UK) was performed on the products. The microstructure of the samples was also analyzed by high-resolution transmission electron microscope (TEM, JEM-2100 UHR, JEOL, Tokyo, Japan) equipped with energy dispersive X-ray spectroscopy (EDS, IET 200, Oxford Instruments, Oxford, UK).

## 3. Results and Discussion

### 3.1. XRD Analysis

**Figure 1:**
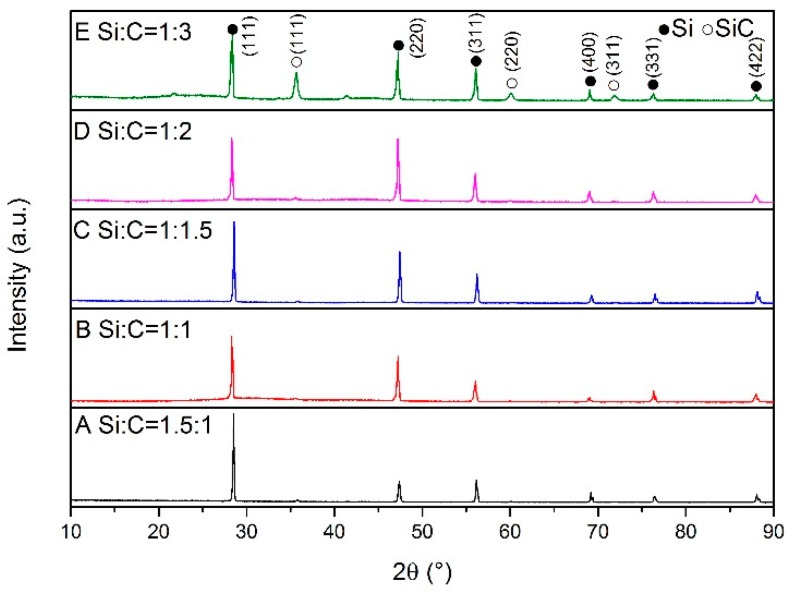
XRD patterns of samples with different Si/C ratios synthesized at 1350 °C.

**Figure 2:**
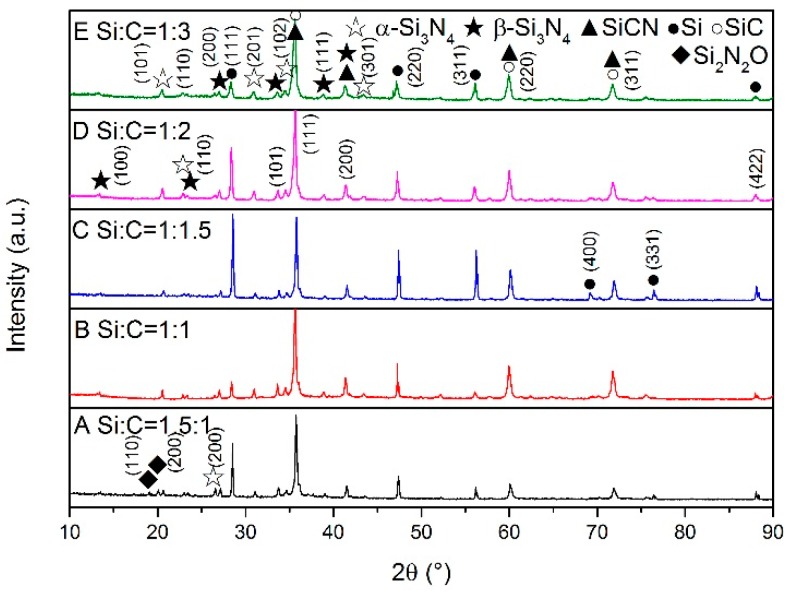
XRD patterns of samples with different Si/C ratios synthesized at 1450 °C.

**Figure 3:**
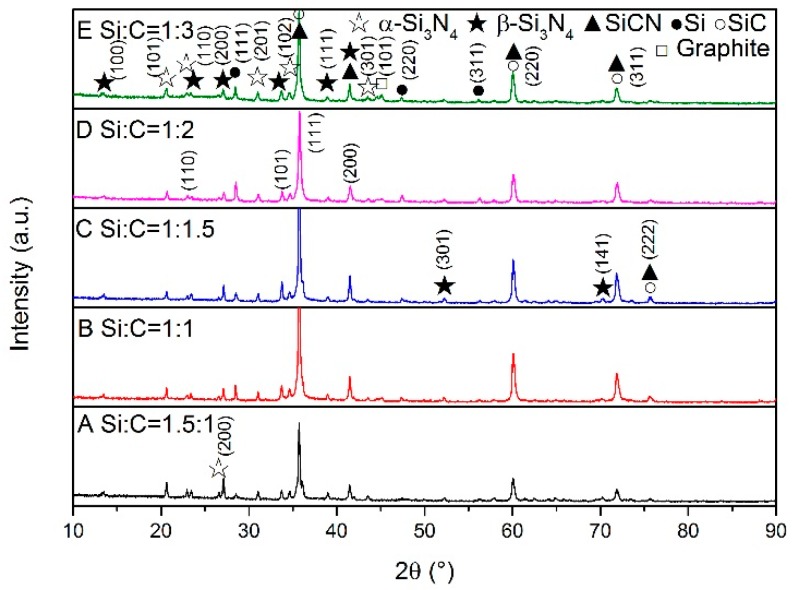
XRD patterns of samples with different Si/C ratios synthesized at 1550 °C.

**Figure 4:**
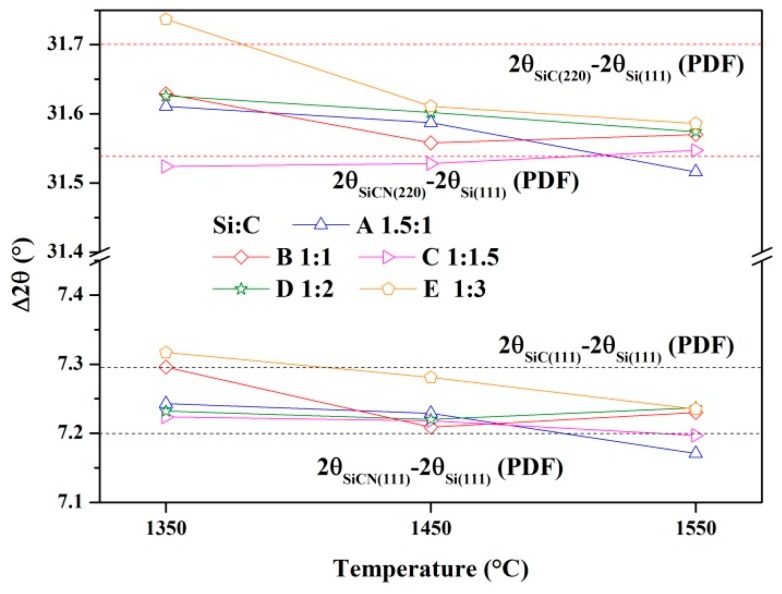
SiC (220) and (111) peaks’ shift of samples synthesized at different temperatures.

XRD patterns of the samples synthesized at different temperatures are shown in Figure 1, Figure 2 and Figure 3. At the lower temperature (1350 °C), amorphous carbon produced from pyrolysis of the cornstarch began to react with silicon as shown in Figure 1. The peaks of SiC can be seen in sample E. When the synthesis temperature was increased to 1450 °C, silicon melted and became a liquid, which was more benefit to the reactions with carbon and N_2_. From Figure 2, it can be seen that the products were mainly composed of SiC (PDF 074-2307), α-Si_3_N_4_ (PDF 083-0700), β-Si_3_N_4_ (PDF 082-0698), SiCN (PDF 074-2308) and unreacted silicon (PDF 077-2108). A small amount of Si_2_N_2_O (PDF 083-1852) existed in sample A because more oxygen was involved in the reaction through silicon powders with a higher proportion in the raw materials. However, Si_2_N_2_O vanished in the sample A after heated at 1550 °C (Figure 3). The diffraction peaks of unreacted silicon in the products became very weak with the consumption in the reactions including carbonization, nitridation and carbonitriding. There was the peak corresponding to graphite in sample E with the excess carbon. The identification of SiCN and SiC will be discussed in the following section. The characterization and testing of samples after firing at 1550 °C were discussed to avoid the influence of Si_2_N_2_O. The shifts of SiC (220) and (111) peaks are shown in Figure 4. The Si (111) peak was selected as the reference. With increasing temperature, SiC peaks basically gradually shifted to the lower degree comparing with the difference values from powder diffraction files, which indicated that nitrogen dissolved into SiC.

### 3.2. XPS Analysis

**Figure 5:**
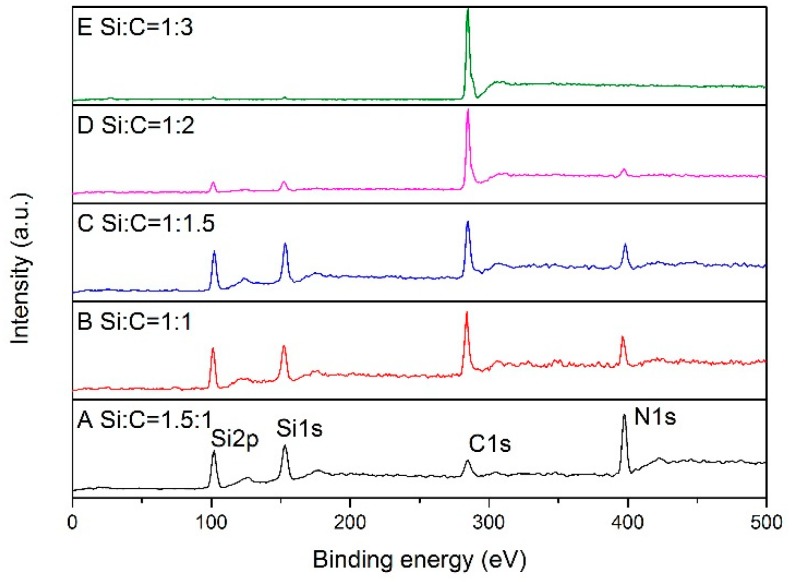
XPS wide scan spectra of samples with different Si/C ratios synthesized at 1550 °C.

The reactions proceeded with a gradually rising temperature. Therefore, XPS analysis was carried out on samples synthesized at 1550 °C. Figure 5 shows XPS wide scan spectra of samples with different Si/C ratios synthesized at 1550 °C. In order to quantify the specific component changes, the area of each element peak and its influencing factors were analyzed quantitatively by the formula.

(1) $$C_{x}=\frac{I_{x}/S_{x}}{\sum_{i}I_{i}/S_{i}}$$

*C_x_* is the percentage of different elements’ atoms in various binding states, *x* is the binding state of elements, *i* is all the atoms in tested elements, *I* is the peak area corresponding to the binding energy, and *S* is the sensitive factor of elements. *S_Si_*_2*p*_ = 0.339, *S_C_*_1*s*_ = 0.296, *S_N_*_1*s*_ = 0.477.

The calculation results are listed in the Table 2. The carbon content in the products basically increased with the ratio of C/Si increasing. The content of N element in the products mainly depended on the proportion of silicon in the starting materials. It was demonstrated that the reaction between raw materials and nitrogen was likely to happen between N_2_ and liquid silicon at the surface.

**Figure 6:**
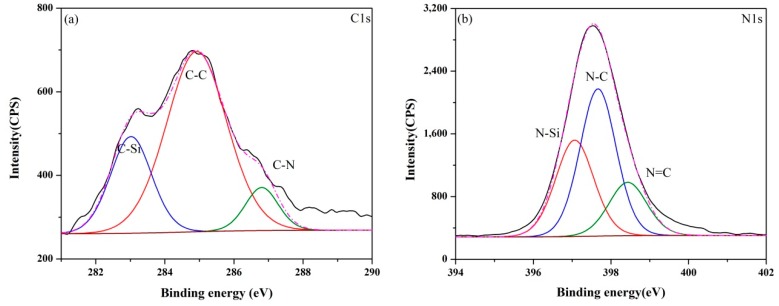

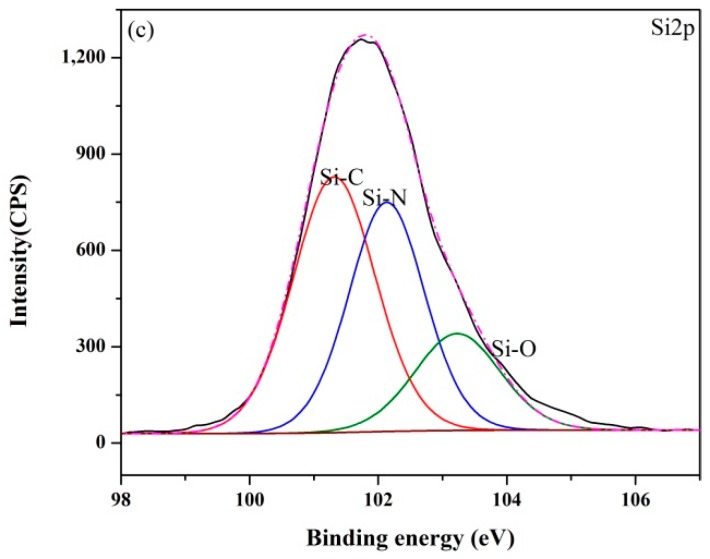
XPS core-level spectra of sample A synthesized at 1550 °C: (**a**) C1s, (**b**) N1s and (**c**) Si2p.

**Figure 7:**
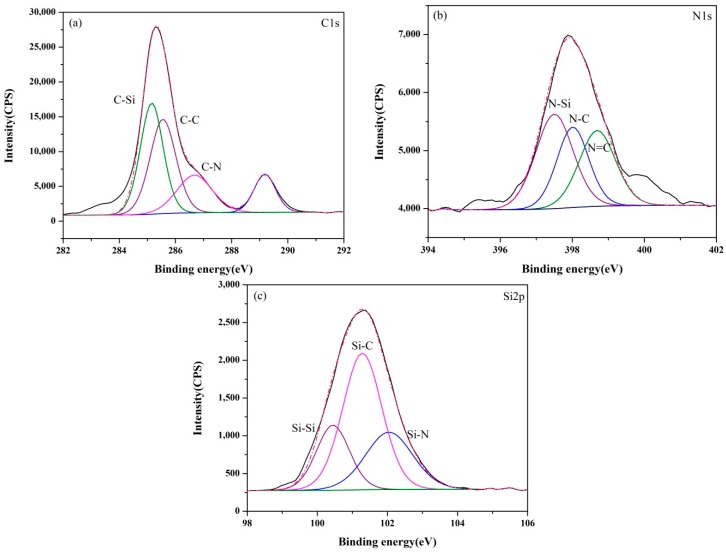
XPS core-level spectra of sample B synthesized at 1550 °C: (**a**) C1s, (**b**) N1s and (**c**) Si2p.

**Figure 8:**
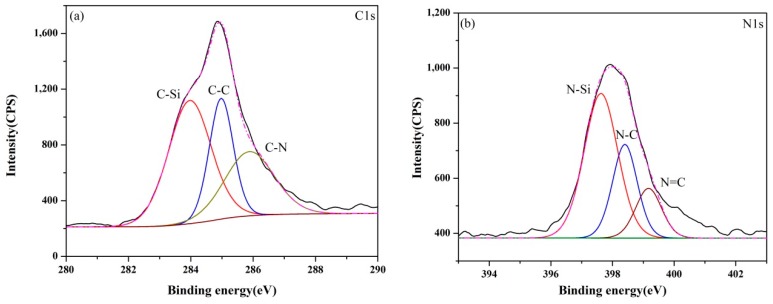

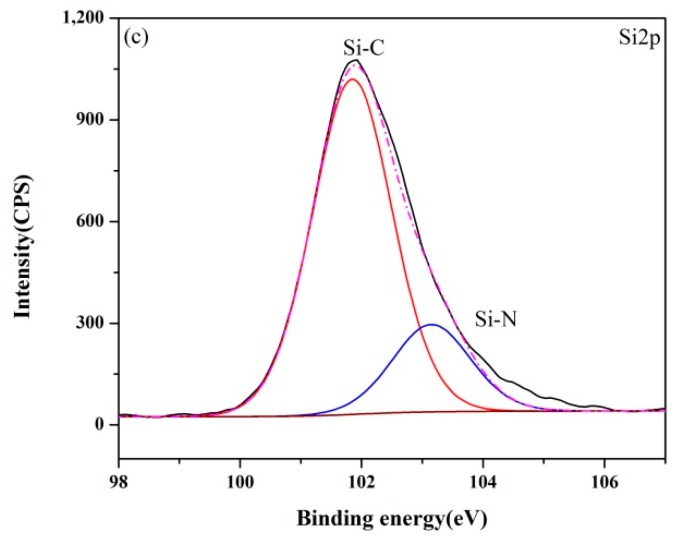
XPS core-level spectra of sample C synthesized at 1550 °C: (**a**) C1s, (**b**) N1s and (**c**) Si2p.

**Figure 9:**
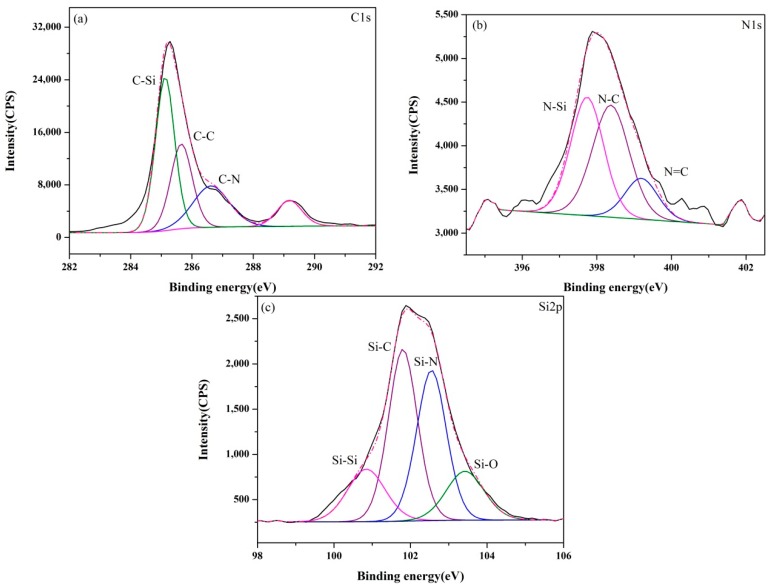
XPS core-level spectra of sample D synthesized at 1550 °C: (**a**) C1s, (**b**) N1s and (**c**) Si2p.

**Figure 10:**
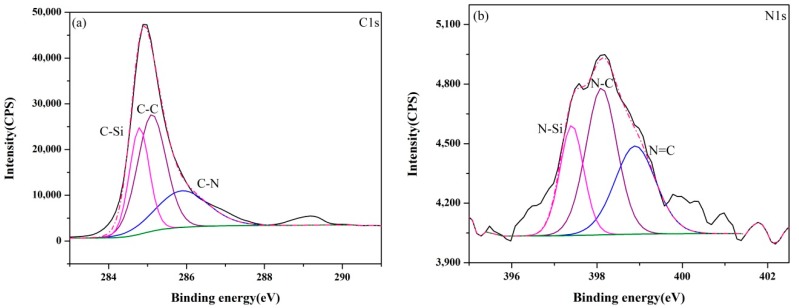

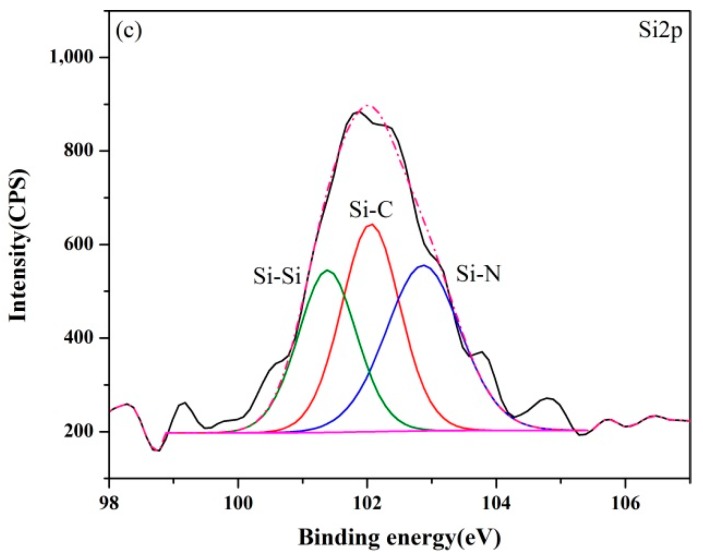
XPS core-level spectra of sample E synthesized at 1550 °C: (**a**) C1s, (**b**) N1s and (**c**) Si2p.

The XPS core-level spectra of samples (A–E) synthesized at 1550 °C are presented in Figure 6, Figure 7, Figure 8, Figure 9 and Figure 10. The information about the chemical bonding of the products was obtained from the scans of C1s, N1s and Si2p. From the deconvolution of the C1s peaks, there were two main components at ~284.4 and ~285.3 eV corresponding to C-Si and C-C bonds, respectively and one minor component at ~286.3 eV attributed to C-N bonds [44]. N1s peaks were composed of N-Si bonds in Si_3_N_4_ and SiCN, and N-C and N=C bonds in SiCN. For Si2p core-level spectra, the broad peaks were identified as a convolution of Si-C and Si-N bonds as well as Si-Si and Si-O bonds as the consequence of unreacted silicon and the oxidation of silicon. 

The XPS spectra of all samples presented similar signatures. However, it was noticed that the fraction of C-N bonds in C1s spectra and N-C/N=C bonds in N1s spectra varied with the ratio of Si/C in the raw materials as shown in Table 3 and Table 4. It was demonstrated that the carbon content had a great influence on the bond between N and Si elements in products. Compared with the monotonic relation between the Si content in raw materials and the nitrogen content in products, the effect of carbon on the change of chemical bond ratio in products was not simply increasing or decreasing. It can be supposed that the proportion of bonds derived from SiCN such as C-N, N-C and N=C represented for the amount of SiCN. C-C bonds in products were as the results of the excess carbon from the pyrolysis of the cornstarch. When the Si/C ratio was less than 1:2, the proportion of C-C bonds decreased with the Si/C ratio, indicating that the amorphous carbon has no influence on the subsequent nitridation reaction. The percentage of N-Si bonds reached the maximum value in sample C with the Si/C ratio of 1:1.5. Though the N content in sample A determined from the wide scan spectrum was highest, Si_3_N_4_ was the major reason according to the XRD results. SiCN may generate by the reaction between Si_3_N_4_ and amorphous carbon at high temperatures. Thus, the relative content of SiCN in samples B and C was higher, even the N contents in them were lower than sample A. When the proportion of carbon in raw materials increased further, the percentage of C-C bonds in products became greater. In contrast, the percentage of N-Si bonds in products declined.

### 3.3. SEM Analysis

**Figure 11:**
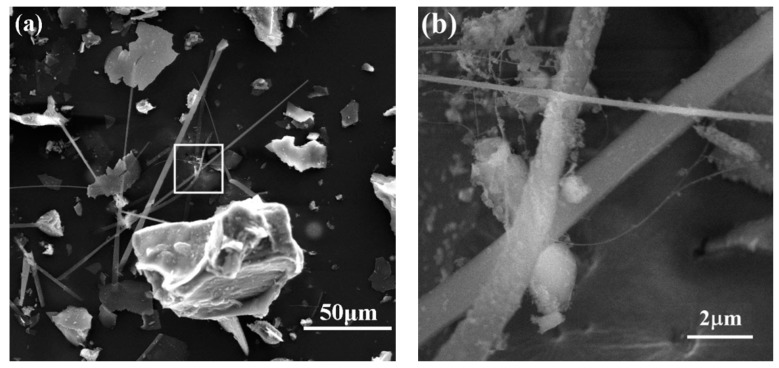
SEM images of samples B synthesized at 1550 °C (Si:C = 1:1): (**a**) Fibers and particles, (**b**) Enlarged view of selected area in (a).

**Figure 12:**
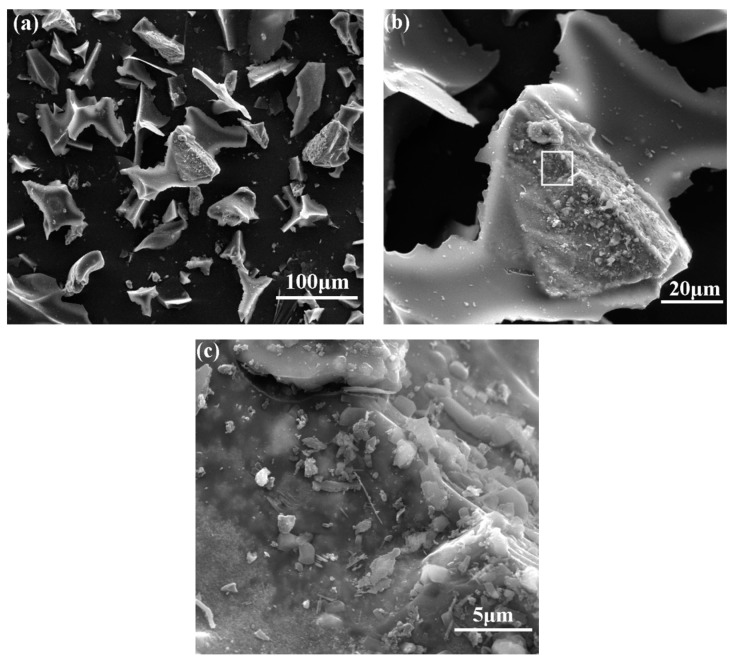
SEM images of samples E synthesized at 1550 °C (Si:C = 1:3): Enlarged view in sequence of (**a**–**c**).

The microstructure of sample B synthesized at 1550 °C is shown in Figure 11. There were fiber-like phases except for the irregular particles in products (seen in Figure 11a). From the image with higher magnification (Figure 11b), it can be seen that the diameter of fibers was around 1 µm. EDS results reveal the presence of Si, C and N in fibers. In contrast, it was hard to find the fiber-like phase in sample E with the higher ratio of C/Si from Figure 12. Comparing sample B with E, the proportion of particulate phase increased with the decrease of Si/C ratio.

### 3.4. TEM Analysis

**Figure 13:**
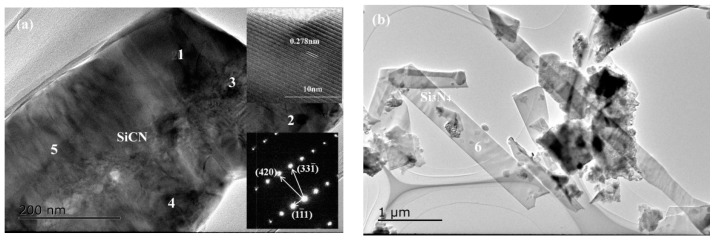
TEM images and electron diffraction patterns of sample A synthesized at 1550 °C (Si:C = 1.5:1): (**a**) SiCN and (**b**) Si_3_N_4_.

**Figure 14:**
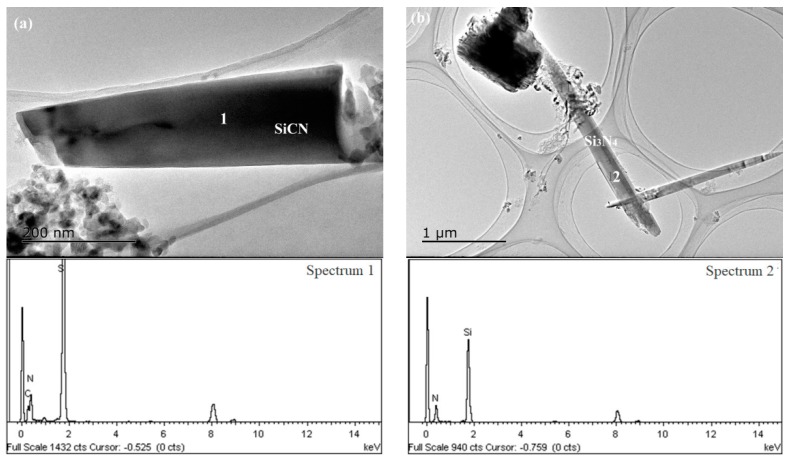
TEM images and EDS patterns of sample B synthesized at 1550 °C (Si:C = 1:1): (**a**) SiCN and (**b**) Si_3_N_4_.

**Figure 15:**
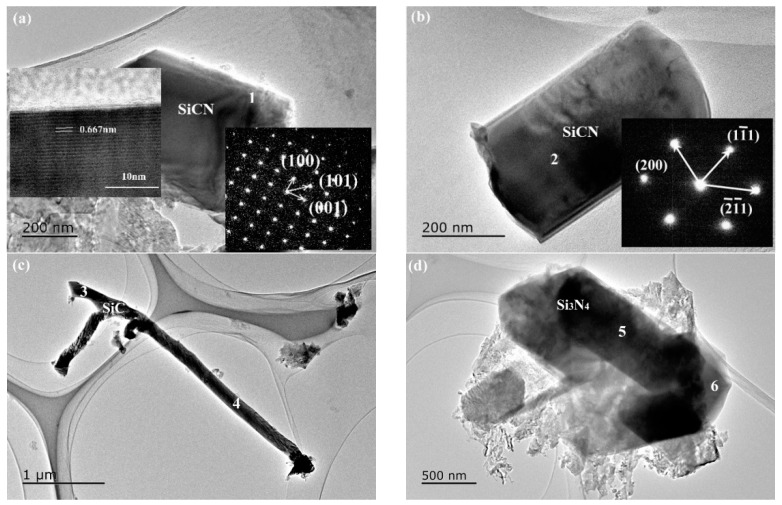
TEM images and electron diffraction patterns of sample C synthesized at 1550 °C (Si:C = 1:1.5): (**a**,**b**) SiCN,(**c**) SiC and (**d**) Si_3_N_4_.

TEM investigation revealed the morphology and the crystal structure of phases in products synthesized at 1550 °C (Figure 13, Figure 14 and Figure 15 and Table 5 and Table 6). For sample A, the chemical composition of the regular particle phase varied with the location as shown in Table 5. The C content of different regions was in the range from 17.1 to 50.9 at%. And the other element N, the content varied between 10.1 and 32.6 at%. In addition, the N content of regions 2 and 4 was much higher than that of regions 1 and 5. The difference of the element distribution in the same particle indicated that SiCN formed by the mechanism, which was that nitrogen atom substituted carbon in SiC [45]. However, the crystal structure of this particle was identified as cubic by electron diffraction (Figure 13a). It can be considered as the result of SiC nitriding. The nitrogen content varies according to the degree of reaction. The belt-like phase with the length of several µm seen in Figure 13b was confirmed as Si_3_N_4_ by EDS (region 6). The length of SiCN was less than that of Si_3_N_4_ in comparison (Figure 14a). The morphology of Si_3_N_4_ was changed from belt to fiber in sample B with equimolar amounts of silicon and carbon (Figure 14b). Rod-like SiCN with the diameter of less than 500 nm was generated in sample C (Figure 15a,b). The N content of SiCN determined by EDS was comparable to that in sample A (Table 6). The formation of cubic and hexagonal SiCN was as results of the nitridation of SiC and the carbonization of Si_3_N_4_. Meanwhile, fiber-like SiC and rod-like Si_3_N_4_ with a relative larger diameter existed in products (Figure 15c,d). In general, the Si contents of SiCN synthesized in this work were higher than those obtained by the vapor deposition and the polymer-derived process [1,46]. The N contents of SiCN prepared by carbonitriding were less than SiCN crystals grown by microwave plasma-enhanced chemical vapor deposition. For the C contents, crystalline SiCN in as-received Si-C-N powders was equal to or less than amorphous SiCN thin film. With the decrease of the Si/C ratio, the surface of liquid silicon was covered with amorphous carbon generated by cornstarch pyrolysis. The nitridation and carbonization reactions strongly depended on the contact area between reactants and atmosphere. So, further study is needed in order to prepare single phase SiCN.

## 4. Conclusions

The Si-C-N powders were synthesized by carbonitriding of the compacts of silicon and cornstarch with Si/C ratios in the range from 1.5:1 to 1:3 in a nitrogen atmosphere. The products were mainly composed of crystalline SiCN, SiC and Si_3_N_4_. The results and analysis can be summarized as follows: The introduction of nitrogen in products mainly relied on the reaction of liquid silicon and N_2_. The N content in products declined with decreasing Si content in raw materials. The low carbon content in raw materials led to insufficient carbon for the carbonization of Si_3_N_4_. It was demonstrated that the Si/C ratio had a great influence on the bonds between N and other elements. With the carbon content increasing, the dominant reaction process was converted from nitridation to carbonation. Rod-like Si-rich crystalline SiCN formed, and fiber-like SiC was generated as well. However, the morphology of Si_3_N_4_ was followed by belt, fiber and rod. The content of SiCN reached the maximum in products synthesized by the compacts with the Si/C ratio of 1:1 and 1:1.5.

## Figures and Tables

**Table 1:** The silicon-carbon ratio of each batch (molar ratio).

Sample	A	B	C	D	E
Si:C	1.5:1	1:1	1:1.5	1:2	1:3

**Table 2:** The element contents in samples calculated from XPS spectra.

Sample	Peak Area (CPS·eV)			Relative Amount (at %)		
	Si	C	N	Si	C	N
A	2870	1493	4727	36.2	21.5	42.3
B	15,139	24,409	5436	32.2	59.5	8.3
C	2083	3394	1240	30.4	56.7	12.9
D	5064	46,429	3634	8.3	87.4	4.3
E	1438	52,586	1631	2.4	95.8	1.8

**Table 3:** The percentage of chemical bonds in C1s (%).

Si/C Ratio	C-Si	C-C	C-N
A 1.5:1	22.8	58.6	18.6
B 1:1	40.2	38.7	21.1
C 1:1.5	36.0	36.5	27.5
D 1:2	57.1	30.0	12.9
E 1:3	28.7	45.5	25.8

**Table 4:** The percentage of chemical bonds in N1s (%).

Si/C Ratio	N-Si	N-C	N=C
A 1.5:1	33.3	48.3	18.4
B 1:1	39.6	28.8	31.6
C 1:1.5	57.2	27.4	15.4
D 1:2	41.7	43.9	14.4
E 1:3	24.2	42.4	33.4

**Table 5:** EDS analysis results of samples (at %).

Atomic (%)	Si	C	N
1	39.0	49.7	11.3
2	50.3	17.1	32.6
3	46.4	32.9	20.7
4	44.9	27.8	27.3
5	39.0	50.9	10.1
6	56.4	—	43.6

**Table 6:** EDS analysis results of samples (at %).

Atomic (%)	Si	C	N
1	56.9	24.4	18.6
2	47.9	32.9	19.2
3	79.6	20.4	—
4	71.0	29.0	—
5	83.9	—	16.1
6	65.8	—	34.2
